# Current Approaches to Neoadjuvant Immunotherapy in Resectable Non-small Cell Lung Cancer

**DOI:** 10.1007/s11912-023-01430-4

**Published:** 2023-05-30

**Authors:** Jay Parekh, Kaushal Parikh, Joshua E. Reuss, Alex Friedlaender, Alfredo Addeo

**Affiliations:** 1 Yale New Haven Health System, Bridgeport Hospital, Bridgeport, CT USA; 2 Mayo Clinic, Rochester, MN USA; 3 Georgetown Lombardi Comprehensive Cancer Center, Washington, DC USA; 4 Clinique General Beaulieu, Geneva, Switzerland; 5 University Hospital Geneva, Geneva, Switzerland

**Keywords:** NCSCL, Neoadjuvant treatment, Early lung cancer

## Abstract

**Purpose of Review:**

For decades, early-stage resectable non-small cell lung cancer (NSCLC), while potentially curable, has been marred by unacceptably high recurrence rates.

**Recent Findings:**

Anti-PD(L)1 immune checkpoint blockade (ICB) has revolutionized the treatment of advanced NSCLC, and with recent approvals in the peri-operative space, is now poised to transform the systemic treatment paradigm for localized and locally-advanced NSCLC.

**Summary:**

In this review, we focus on neoadjuvant ICB in resectable NSCLC, highlighting the pre-clinical rationale for neoadjuvant ICB, early clinical trials, randomized phase 3 trial data, and future directions for resectable NSCLC.

**Graphical Abstract:**

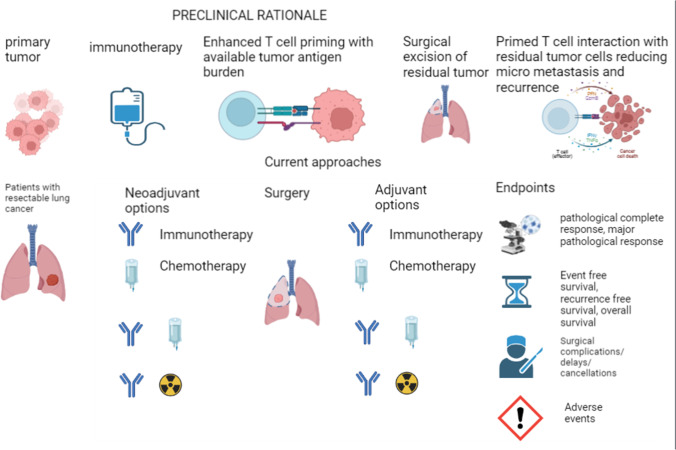

## Introduction

According to American Cancer Society 2023 statistics, more than 127,000 people are estimated to die of lung cancer in the US [1]. Although there has been a decline in lung cancer incidence and mortality over the last couple of decades, the 5-year survival rate for non-small cell lung cancer(NSCLC) is just over 25% [1, 2].Increased uptake of lung cancer screening has helped in diagnosing more patients with earlier stages of lung cancer [3]. Surgery remains the primary treatment modality among early stage NSCLC,as well assome cases of stage IIIA NSCLC [4]. Multiple randomized trials have shown survival benefit with the addition of adjuvant cisplatin-based doubletchemotherapy in patients who underwent surgical resection for early-stage lung cancer (stage IB-IIIA) [5, 6].The IALT trial showed a roughly 4% absolute difference in 5-year survival rates with 3 to 4 cycles of adjuvant cisplatin-based therapy after resection. Similarly, the ANITA trial demonstrated an absolute improvement by 8.4% in survival rates with adjuvant chemotherapy compared to observation after prolonged follow-up at 7 years. The LACE meta-analysis of over 4000 patients showed a 5.4% improvement in OS with adjuvant chemotherapy [7]. In parallel, neoadjuvant chemotherapy has also been studied in resectable NSCLC.Preclinically, in 1987, mice models with Lewis lung carcinoma treated with cyclophosphamide and ifosfamide prior to surgery demonstrated improved survival compared to those who underwent surgery alone [8]. Subsequently, early studies with neoadjuvant chemotherapy were based on the rational that after surgical resection, blood supply to the tumor may be compromised hence adjuvant therapy delivery may be impaired [9]. These studies demonstrated an improvement in surgical complete resection rates, improved local control and survival [9]. A metanalysis of 13 trials showed that addition of neoadjuvant chemotherapy has a significant survival benefit compared to surgery alone but individual studies have demonstrated inconsistency,reducing the confidence in this option since it comes along with adverse events and possibility of delay in surgery [10].To date, there have not been any randomized trials in NSCLC showing benefit of one perioperative treatment over the other. The phase III NATCH trial randomized 624 patients to receive neoadjuvant chemotherapy (*n*=201), adjuvant chemotherapy (*n*=211) or surgery alone (*n*=212). Five year survival rates were 46.6%, 45.5%, and 44% across neoadjuvant, adjuvant and surgery alone arms, respectively with no statistically significant difference in outcomes [11]. The investigators did note that more patients were able to receive preoperative therapy than adjuvant therapy. Another meta-analysis using individual-participant data from 15 randomized controlled trials showed an 5% absolute benefit with preoperative chemotherapy on 5-year survival compared to observation, but no difference between preoperative and postoperative therapy [12]. Five-year recurrence-free survival (RFS) with preoperative chemotherapy ranged between 30% and 36% [12]. Immune checkpoint inhibitors (ICIs) have changed the treatment landscape and improved outcomes of patients with metastatic NSCLC and are now becoming standard of care in early-stage disease too. In this review, we will discuss the clinical evidence supporting the use of immunotherapy in neoadjuvant treatment of NSCLC. We will discuss the rationale behind neoadjuvant immune checkpoint blockade and early clinical data. We provide an in-depth review of current phase III evidence supporting neoadjuvant immunotherapy. We conclude by discussing ongoing studies, future strategies of combining neoadjuvant and adjuvant immunotherapies, and the use of circulating tumor markers to predict outcomes.

## Rationale for Neoadjuvant Immunotherapy

Neoadjuvant therapy may be useful in debulking or downstaging tumors prior to surgery, improving surgical resectability of borderline tumors [13] (Table 1). The rationale for immunotherapy in the neoadjuvant setting was based on the hypothesis that the high tumor antigen burden may be helpful in T cell priming. This presence of tumor antigens can induce T cell clonal expansion which has been shown to predict better survival in patients with melanoma treated with immune checkpoint inhibition [14]. T cell priming may help to eliminate micrometastatic disease after surgical resection of primary tumor and thus reduce recurrence, besides reducing the size of the primary tumor [15]. This anti-tumor activity may also be a mechanism for limiting spread in tumor draining lymph nodes where antigen presentation to T cells occurs. A 2016 preclinical study of mice inoculated with triple negative breast cancer cell lines showed improved survival and micrometastatic disease eradication with neoadjuvant anti-PD1 inhibition compared to adjuvant anti-PD1 inhibition. A higher level of CD8+ T cells and interferon gamma were also found among the mice group with better survival receiving neoadjuvant blockade [16].They also observed presence of tumor-directed CD8+ T cells up to 170 days out of tumor inoculation in the neoadjuvant group. Along similar lines, murine NSCLC models were treated neoadjuvantly or adjuvantly withanti-PD1 monotherapy, anti-CTLA4 monotherapy or the combination. Mice with combined neoadjuvant therapy had a prolonged survival and reduced distant recurrence compared to mice with adjuvant combined therapy. Moreover, neoadjuvant therapy was associated with enhanced tumor infiltrating lymphocytes (TILs) in the resected primary tumor, as well as in lung metastases [17].Using the cancer genome atlas (TCGA), McGranahan et al. observed that early-stage lung adenocarcinomas with high clonal neoantigen burden were associated with improved anti-tumor T-cell gene expression, immune-related gene expression, inflamed tumor microenvironment (TME), and survival [18]. Thus, the hypothetical rationale and preclinical evidence suggest a potential benefit of neoadjuvant immunotherapy in early-stage lung cancer.

**Table 1:** Neoadjuvant therapy

Pros	Cons
Higher antigen load for more robust immune response compared to adjuvant immunotherapy	Delay in surgical resection
Patient performance status more suited for completing treatment	Lack of long-term survival evidence
Less concern for toxicity compared to cytotoxic chemotherapy	Adverse effects may lead to cancellation of surgery, hospitalization, and death
Increased rates of R0 resection	Concern for fibrosis, surgical complications
In-depth tumor and TME assessment following resection	Progression of disease precluding definitive resection

## Neoadjuvant Immunotherapy in NSCLC

Early evidence for neoadjuvant immunotherapy came from a pilot study of 21 patients with resectable stage I-IIIA NSCLC, who received two doses of preoperative nivolumab followed by definitive surgery. The study reported no delay in surgery due to treatment related events and only 1 grade 3 event of pneumonitis was observed but this patient underwent uncomplicated surgical resection. The median interval reported between last dose of nivolumab and surgery was 18 days. Although merely 2 patients had a radiographic partial response (10%), major pathologic response (less than 10% viable tumor cells at resection—MPR) of 45% was observed and 2 patients had a complete pathologic response (pCR) [19]. At a median 12-month post-operative follow-up,the rate of RFS was 80%.More recently, 5-year survival outcomes of this study were reported, and RFS and OS rates were 60% and 80% [20]. Particularly, 89% of patients who had MPR were alive and disease-free at 5 years. The investigators conducted whole exome sequencing from pretreatment tumors of 12 patients. A higher mean mutational burden was associated with MPR but was not associated with improved RFS or OS. Deep sequencing of T cell receptor-βchain CDR3 regions demonstrated a higher frequency of tumor specific clonal T cell population within tumor and peripheral blood among patients with major pathological response. Besides demonstrating safety, this pilot study also provided valuable evidence of immune mechanisms for anti-tumor activity of neoadjuvant nivolumab.

Lung Cancer Mutation Consortium 3 (LCMC3) study was a single arm phase II trial of 2 doses of neoadjuvant atezolizumab followed by adjuvant therapy at investigators’ discretion. In the primary efficacy population of 149 patients with resectable stage IB to IIIB NSCLC without activating EGFR or ALK alterations, MPR was 20% and 3-year survival was 80%. Of note, no radiological or major pathological responses were observed among tumors with EGFR or ALK alterations [21]. Another ongoing phase 2 trial of one dose of neoadjuvant atezolizumab (PRINCEPS) presented interim results and reported MPR in 4 out of 30 patients with stage IA to IIIA disease [22].Dual immune checkpoint blockade with PD1/PDL1 and CTLA4 inhibitors has also been studied. Reuss et al. terminated their single arm study of neoadjuvant ipilimumab and nivolumab after observing treatment related adverse events (TRAEs) in 6 out of 9 enrolled patients and grade 3 or higher adverse events (AEs) in 33% patients [23]. The phase II NEOSTAR trial by Cascone et al randomized 44 patients with stage IB-IIIA NSCLC to receive neoadjuvant nivolumab (*n*=23) or nivolumab-ipilimumab (*n*=21). The rates of MPR were 22% and 38% for nivolumab and nivolumab-ipilimumab, respectively. Interestingly, 2 patients in nivolumab arm achieved pCR compared to 6 patients in the doublet immunotherapy arm [24]. The phase II NeoCOAST trial studied neoadjuvant single agent durvalumab as well as its combinations with oleclumab (anti-CD73 antibody), monalizumab (anti-NKG2A antibody) and danvatirsen (anti-STAT3 antisense olignoucleotide) in patients with stage I-IIIA NSCLC and observed MPR rates of 11.1%, 19.0%, 30.0%, and 31.3%, respectively as well as pCR rates of 3.7%, 9.5%, 10.0%, and 12.5%, respectively [25]. Further analysis revealed that pathologic responses were not associated with TMB, and combination immunotherapy regimens led to a more inflamed TME and greater upregulation of inflammatory gene signature than single agent durvalumab [26]. These studies have laid the foundation for a promising decade of paradigm changing phase III trials involving immunotherapy in neoadjuvant setting.

## Neoadjuvant Immunotherapy with Radiation Therapy

There have been several reports with evidence that radiation therapy (RT) not only works by inducing DNA damage but also, cancer cell death causes release of antigens which can induce a proinflammatory state and this can be used synergistically with immunotherapy to obtain better efficacy [27]. In preclinical models, combination RT and anti-CTLA4 therapy showed reduction in primary tumor size. Further, the anti-tumor activity of anti CTLA 4 therapy was significantly increased with combination RT in secondary tumors which did not receive RT, supporting the rationale of abscopal effect [28]. This study as well as several other studies have shown an increase in CD8+ TILs, PDL-1 expression in tumors treated with RT based regimens [29, 30]. Based on the hypothesis that SBRT might be a potent immunomodulator, it was tested along with neoadjuvant immunotherapy. A phase 2 trial among patients with stage I to IIIA NSCLC documented no major difference in operative and postoperative outcomes with combination durvalumab and RT [31]. The trial had a durvalumab monotherapy arm, and an arm with 3 fractions of 8Gy SBRT followed by 2 cycles of durvalumab. Sixteen out of 30 patients achieved MPR in the SBRT plus durvalumab arm, while only 2 out of 30 patients in durvalumab monotherapy arm achieved MPR [32].There were no treatment related deaths within 30 days and the TRAE profile appeared relatively similar in both arms. Currently, several other ongoing trials are evaluating the safety and efficacy of concomitant RT with neoadjuvant immunotherapy [33, 34]. A small phase 1 study combining neoadjuvant pembrolizumab with concurrent chemoradiation had to be stopped early due to 2 grade 5 events and 7 serious events [35].

## Combination of Neoadjuvant Immunotherapy and Chemotherapy

Mechanistically, the chemotherapy is supposed to cause cell death, releasing cancer neoantigens which can lead to cytotoxic T cell expansion under the influence of immunotherapy. The combination of pre-operative chemotherapy and immunotherapy was first reported by Yi et al. in a phase II study of 24 patients with stage IB-IIIA NSCLC treated with carboplatin, paclitaxel and ipilimumab [36]. The primary objective of this study was to assess increase in tumor antigen specific T cell expansion after neoadjuvant therapy. Ipilimumab was associated with higher CD4+ and CD8+ T cell activation in blood as well as in the TME but did not lead to increase in tumor-associated antigen specific T cell activation. Subsequently, Shu et al. enrolled 30 patients with stage IB-IIIA NSCLC on a single arm, phase II trial of four cycles of neoadjuvant carboplatin, nab-paclitaxel, and atezolizumab followed by surgery. Seventeen out of 30 patients (55%) achieved MPR and 10 patients (33%) achieved pCR. Notably, 23 patients in this trial had stage IIIA disease, of which 6 achieved pCR. Similarly,19 patients had pathologically confirmed N2 disease, of which 11 had nodal clearing [37]. This further underlines the hypothesis that treating larger tumors may produce a more robust immune response. Around the same time, Spanish investigators studied neoadjuvant chemoimmunotherapy with carboplatin, paclitaxel and nivolumab for three cycles followed by one year of adjuvant nivolumab in patients with stage IIIA NSCLC in the phase II NADIM trial[38]. Twenty-five (54%) of the 46 patients enrolled in this trial had multi-station N2 nodal involvement and another 9 (20%) had single-station N2 nodal involvement. They reported remarkable results with 34 of 41 patients who underwent surgery achieving MPR (83%) and 26 of them achieving pCR (63%). Like several of the above studies, no definite correlations could be inferred between PDL1 expression or TMB and pathologic responses. These studies provided early clinical evidence of combining chemotherapy with immune checkpoint inhibitors in resectable NSCLC. Its follow up trial, NADIM II, randomized patients with stage IIIA NSCLC to receive the above mentioned chemoimmunotherapy or platinum-doublet chemotherapy neoadjuvantly and showed that the addition of nivolumab markedly improved pCR rates (36.2% vs 6.8%; RR 5.25, *p*=0.071), MPR rates (54% vs 14%) and objective response rates (ORR; 74% vs 48%) [39].

Perhaps, the strongest evidence for neoadjuvant chemoimmunotherapy use currently comes from the CHECKMATE 816 study. This large phase III trial randomized 358patients with stage IB-IIIA NSCLC to receive three cycles of preoperative nivolumab plus platinum-based chemotherapy (*n*=179) or chemotherapy alone (*n*=179) followed by surgery. The primary endpoints of this trial were event-free survival (EFS) and pCR rates, while secondary endpoints included MPR, time to death or distant metastasis, and overall survival (OS). The triplet arm led to pCR rates of 24.0% compared with 2.2% with chemotherapy (OR 13.94, 95% CI 3.49–55.75; *p*<0.001). Similarly, MPR rates were also significantly higher with adding nivolumab to chemotherapy compared to chemotherapy alone (36.9% vs 8.9%; OR 5.70, 95% CI 3.16–10.26). Rate of pCR was higher in the chemoimmunotherapy arm across various subgroups of PDL1 expression, disease stage, tumor histology, and TMB. The trial also met its other primary endpoint of EFS. Median EFS was significantly longer with chemoimmunotherapy (31.6 months vs 20.8 months; HR 0.63; 97.38% CI 0.43–0.91; *p*=0.05). The magnitude for EFS benefit with triplet therapy was more profound with stage IIIA disease than earlier stages. Given the larger sample size, some inference could be made from the subgroup analysis which also showed greater benefit of nivolumab and chemotherapy in patients with PDL1 expression of 1% or greater and in non-squamous histology, in line with mature advanced stage data. More importantly, the safety analysis revealed similar grade 3 or 4TRAEs with chemoimmunotherapy and chemotherapy (33.5% and 36.9%).Based on these results, the US FDA, and more recently UK’s NICE approved neoadjuvant chemotherapy and nivolumab for treatment of patients with resectable NSCLC [40, 41].

It is now well known that ICIs have minimal efficacy in NSCLC with EGFR mutations, which are highly prevalent in NSCLC in Asia [42–45]. To limit the impact of EGFR mutations on efficacy of neoadjuvant ICI, Shen et al. investigated2 cycles of neoadjuvant chemoimmunotherapy followed by surgery in patients with stage IIB-IIIB squamous cell lung cancer. All 37 patients enrolled in the study completed 2 cycles of pembrolizumab with nab-paclitaxel and carboplatin and underwent R0 resection. pCR, which was the primary endpoint of the study, was reported in 17(46%) patients and 24 (65%) patients had MPR [46].The SAKK network performed a phase II study using cisplatin and docetaxel combined with durvalumab preoperatively among patients with stage IIIA NSCLC with adjuvant durvalumab continued after resection. Sixty-two out of 67 patients received neoadjuvant durvalumab along with chemotherapy. Fifty-five patients underwent resection, of which 51 patients underwent R0 resection. The primary endpoint in this study, 1-yearEFS was 73%. 62% of patients achieved MPR and 18% had pCR [47].

## Surgical Outcomes with Neoadjuvant ICI

Complete surgical resection, without any macro- or microscopic residual disease (R0 resection) is the only gold-standard surgical procedure consistently associated with improved survival in early-stage NSCLC. One of the major concerns with neoadjuvant immunotherapy was its effects on immediate surgical outcomes due to possible immunotherapy-related scarring and fibrosis limiting R0 resection. A small case series of 5 patients initially reported feasibility of pulmonary resection among patients who have received neoadjuvant immunotherapy but mentioned dense fibrosis as possible complication of the neoadjuvant therapy [48]. Subsequent small retrospective studies have documented feasibility reporting no major intraoperative complications with neoadjuvant immunotherapy. Surgical outcomes from the pilot study of preoperative nivolumab, described earlier, were comparable with prior evidence. There were no delays to surgery and no operative mortality. Importantly, more than 13 procedures attempted via robotic or video-assisted thoracoscopic approach required conversion to thoracotomy due to hilar inflammation and fibrosis. The median interval between last dose of nivolumab and surgery in this study was 18 days. According to the protocol, patients underwent imaging 7 days prior to surgery to assess for radiological response [49]. In the TOP1501 phase II study of neoadjuvant pembrolizumab, 23 out of 25 patients underwent VATS and 5 of those were converted to thoracotomy, but none were due to increased fibrosis. Twenty-two out of 25 patients had R0 resection and 3 had R1 resection. Encouragingly, no patient had immunotherapy-related pneumonitis or interstitial fibrosis and all patients were alive 90 days post-surgery [50]. A retrospective study among patients with stage IIIA/N2 NSCLC comparing surgical outcomes following neoadjuvant nivolumab and chemotherapy showed that the median interval between neoadjuvant therapy and surgery was not different among the two groups and similar results were obtained in terms of immediate post operative complications [51]. Multiple other small prospective and retrospective studies have shown similar acceptable surgical outcomes without delays or complications. In the NEOSTAR trial, 89% of patients completed surgical resection across both arms with similar complication rates. Thirty and 90-day mortality rates were comparable to historical controls and all patients underwent R0 resection. The median interval between immunotherapy and surgery in this study was 31 days while radiological assessment was planned for at least 2 weeks after last dose [52].

Certainly, the largest surgical outcomes data so far also comes from the CHECKMATE-816 trial. Patients underwent surgery up to 6 weeks after neoadjuvant treatment with a restaging scan performed within 14 days prior to surgery. Numerically, median duration of surgery and minimally invasive procedures were more common in the nivolumab plus chemotherapy groups and pneumonectomies were less common in this group too. 83.2% of patients in the chemoimmunotherapy arm underwent R0 resection compared to 77.8% in the chemotherapy arm. Overall, there is growing body of evidence to dismiss major surgical concerns after neoadjuvant immunotherapy.

## Pathological Outcomes as Surrogate Endpoints for Survival

One major advantage of neoadjuvant therapy clinical trials is the availability of surgical resection specimens to perform pathologic and translational analysis, assess the tumor immune response, and evaluate temporal changes due to therapy. The common pathologic outcomes evaluated include pCR and MPR. Pathological complete response has been evaluated as a surrogate marker in several other cancers including breast cancer and rectal cancer and has been to correlate with overall survival [53, 54]. Multiple chemotherapy-only neoadjuvant trials have found pCR to be an elusive endpoint with results ranging from 0 to 16% [55]. For instance, in Checkmate816, pCR rate was merely 2.2% with chemotherapy. Consequently, major pathologic response (MPR), seen in about 20% of patients with preoperative chemotherapy, was felt to be a more realistic marker to study association with survival outcomes. However, remarkable increase in pCR with ICIs in above mentioned trials has increased interest in this endpoint in NSCLC. In their retrospective analysis, Pataer et al. reported that MPR after neoadjuvant chemotherapy was associated with better OS (*p*=0.005) and DFS (*p*=0.01). Patients with MPR had longer 5-year OS compared to those without MPR (85% vs 40%; *p*<0.0001) and longer 5-year DFS (78% vs 35%; *p*<0.001) [56]. In an aggregate data meta-analysis of over 7000 patients treated with neoadjuvant chemotherapy or chemoradiation, pooled pCR rate was 18%. Patients who achieved pCR had better OS (HR 0.50) and EFS (HR 0.46) than those who did not [57].

In the earlier described neoadjuvant ICI trials, all studies except Checkmate-816 were underpowered for survival analysis. Checkmate-816 has met its two primary endpoints, and in exploratory analyses, EFS appeared to be longer in patients who achieved pCR in both treatment arms. However, the endpoint of most interest is OS. While OS data from Checkmate-816 continue to mature, survival from the earlier single agent nivolumab pilot study showed 5-year OS of 80% and recurrence free survival (RFS) of 60%. Eight out of 9 patients who achieved MPR as well as both patients with pCR were alive and disease free at 5 years [20]. Similarly, EFS has also been analyzed as a surrogate marker in lung cancer. In another meta-analysis of 74 neoadjuvant lung cancer studies, a significant correlation was found between median EFS and OS with a Pearson’s correlation coefficient of 0.819 [58]. More phase III evidence supporting the association of pathologic response with survival will provide confidence in using it as a surrogate marker for survival to develop further trials and for clinical implementation of neoadjuvant ICI. However, we must use caution when extrapolating surrogate endpoints from other cancers and using them in NSCLC without validation.

## Future directions

Perioperative immunotherapy has become standard of care in resectable NSCLC. Currently, there are 3 approved regimens in this scenario—neoadjuvant chemotherapy plus nivolumab; adjuvant atezolizumab from the IMPower010 trial and adjuvant pembrolizumab based on the KEYNOTE-091 study. The latter two trials did not include any neoadjuvant therapy. NADIM II was one of the earliest studies to incorporate neoadjuvant and adjuvant immunotherapy. Patients in the chemoimmunotherapy arm received adjuvant nivolumab for 6 months after resection. The regimen led to a 24 month overall survival of 85.3% in chemoimmunotherapy arm compared to 64.8% in chemotherapy arm [59]. More recently, two press releases noted that the KEYNOTE-671 trial of neoadjuvant chemotherapy and pembrolizumab followed by adjuvant pembrolizumab and the AEGEAN trial with durvalumab in a similar regimen met their primary endpoints and results are expected to be presented at a major oncology conference [60, 61]. This coincided with the publication of the randomized phase II S1801 trial in patients with resectable stage III-IV melanoma [62]. Patients were randomized to receive either 3 cycles of neo-adjuvant followed by surgery and 15 cycles of adjuvant pembrolizumab or surgery followed by 18 cycles of adjuvant pembrolizumab. This study met its primary endpoint and EFS was significantly longer in the neoadjuvant-adjuvant group (*p*=0.004). This is the first trial to head-to-head compare neoadjuvant-adjuvant and adjuvant-only ICI approaches and confirms the added benefit of neoadjuvant ICI over adjuvant ICI. The fact that 3 doses of neoadjuvant ICI so drastically affected outcomes is a remarkable finding, perhaps, the best testimonial to support the rationale behind neoadjuvant ICI trials. Throughout the immunotherapy story, lung cancer treatment has followed the melanoma path and there are valuable lessons to learn from this trial in designing perioperative clinical trials in lung cancer. Currently, there are several ongoing trials evaluating neoadjuvant ICI combinations followed by surgical resection (Table 2). However, several pertinent questions remain unanswered:

**Table 2:** Neoadjuvant ICI combinations followed by surgical resection

Trial	Phase	Drug	Outcome	Comments
Checkmate816[63]	III	Nivolumab + Platinum chemotherapy Vs chemotherapy	31 months EFS in nivolumab plus chemotherapy arm vs 20 months in chemotherapy arm. 24% pCR in nivolumab plus chemotherapy arm vs 2.2% in chemotherapy arm.	Phase 3 trial comparing to neoadjuvant chemotherapy. Both groups were similar in terms of safety data.
NADIM[38]	II	Nivolumab + Chemotherapy	77% PFS at 24 months and 30% grade 3 events but no surgery delays	Single arm phase 2 open label trial
Shu[37]	II	Atezolizumab + Platinum chemotherapy	57% MPR	open-label, multicentre, single-arm, phase 2 trial
SAKK 16/14[47]	II	Durvalumab + Chemotherapy	73% 1 yr EFS and 18% pCR	open-label, multicentre, single-arm, phase 2 trial
NEOSTAR[24]	II	Nivolumab or Nivolumab + Ipilimumab	24% MPR in nivolumab arm and 50% MPR in nivolumab + ipilimumab	Open label, single institution, phase 2 trial
Zhao[64]	II	Toripalimab+ chemotherapy	60.6% MPR including 45.5% pCR	Phase 2 trial in stage 3 NSCLC
LCMC3 trial [21]	II	Atezolizumab	20% MPR and 6 % pCR with 80% 3 year survival rate.	Open label phase 2 trial, excluded patients with EGFR mutation and ALK alterations.
NEOMUN[65]	II	Pembrolizumab	27% major pathological response	Open label, phase 2 trial
TONG[50]	II	Pembrolizumab	88% with R0 resection and 28% with MPR	Open label phase 2 trial
Gao[66]	I	Sintilimab	52% TRAE, 10% Grade 3 TRAE, 40.5% MPR	Open label phase 1b trial
Shen[46]	N/A	Pembrolizumab	45.9% pCR with 64.9% MPR	Single center prospective trial with patients with squamous cell lung cancer were only included to exclude the influence of EGFR mutations of effect of immunotherapy.
Wang[67]	N/A	Nivolumab, Pembrolizumab, Camrelizumab plus chemotherapy	29.1% pCR and 65.2% partial remission	
Dai[68]	N/A	tislelizumab, nivolumab, pembrolizumab, sintilimab plus chemotherapy vs chemotherapy	57.89% vs 5.3% pCR rate and 78.95% vs 10.26% MPR rate	Propensity matched retrospective cohort study.
Dickhoff[33]	II	Ipilimumab plus nivolumab with chemoradiation		Ongoing phase 2 trial in T3-4N0-1 NSCLC patients.
Dong[69]	N/A	Immunotherapy systematic review	Overall MPR effect size of 44% and pCR effect size 22%.	Meta-analysis of 18 studies with 678 patients.
Faehling[70]	N/A	Pembrolizumab, nivolumab plus chemotherapy	MPR was 68% and pCR was 53%. At 24 months overall survival was 87.2%	Retrospective real world cohort study with 59 patients including about 44% with oligometastatic disease.
CANOPY-N[71]	II	Canakinumab, pembrolizumab	ongoing	Phase 2 trial evaluating IL-1beta inhibition
Ge[72]	N/A	Chemotherapy plus immunotherapy Vs immunotherapy metanalysis	MPR of 68% after combination with chemotherapy compared to 39.1% with immunotherapy alone.	Metanalysis of 13 studies comparing immune checkpoint inhibitors alone vs in combination with chemotherapy.
SQUAT Trial[34]	II	Chemoradiation plus neoadjuvant durvalumab and consolidation durvalumab after surgery	Ongoing.	
Jiang[73]	N/A	Neoadjuvant immunotherapy Vs combined chemo-immunotherapy	Neoadjuvant chemoimmunotherapy had MPR of 53.3% compared to 28.6% with single agent neoadjuvant immunotherapy	Meta-analysis included 16 studies and 988 patients.
IONESCU[74]	II	Durvalumab monotherapy	19%MPR with median 12-month DFS of 78%. No serious grade 3 and above events.	multicenter, single-arm, phase II trial
Reuss[23]	Ib/II	Nivolumab plus ipilimumab	6 out of 9 patients in the study had treatment related adverse events and 33% grade 3 or more events. 1 death post operatively due to acute respiratory distress syndrome.	Trial terminated early due to toxicity.

*pCR* pathological complete response, *EFS* event free survival, *MPR* major pathological response, *DFS* disease-free survival, *EGFR* epidermal growth factor receptor, *ALK* anaplastic lymphoma kinase

*Who are the patients likely to benefit from addition of adjuvant immunotherapy after receiving neoadjuvant chemoimmunotherapy?* A potential avenue to study this question is a better understanding of the tumor-immune interaction and the post-ICI TME. There is an evolving role of circulating tumor DNA (ctDNA) beyond diagnostic molecular testing. Molecular residual disease (MRD) is a concept first studied in hematologic malignancies and is increasingly being evaluated in solid organ malignancies. A commonly used approach to detect MRD is identifying ctDNA in the blood following treatment. Detection of ctDNA post-resection has been associated with early recurrence and poor outcomes. In a longitudinal ctDNA assessment study following surgery for resectable NSCLC, patients with detectable ctDNA post-surgery had a shorter RFS than those with undetectable ctDNA (HR 4.0, *p*<0.001) [75]. This was observed regardless of adjuvant therapy receipt; however, among patients with detectable post-surgery ctDNA, those who received adjuvant therapy had a longer RFS than those who did not (*p*<0.05). Interestingly, risk of relapse in ctDNA negative patients did not differ with adjuvant chemotherapy receipt. Therefore, clinical trials standardizing MRD detection assays and incorporating them as a means to escalate or de-escalate therapy may improve patient selection for adjuvant therapy.

*What are the predictors of response to neoadjuvant ICI?* Predictive response markers such as PDL1, TMB, tumor mutational patterns, and smoking status have been extensively studied in advanced NSCLC and have been largely inconclusive [76, 77]. Neoadjuvant clinical trials give us the unique opportunity to investigate the TME in greater depth than possible with small biopsies in advanced disease. A concerted effort in early phase biomarker exploration and studies designed with strong translational research components are needed. Importantly, patients with actionable driver alterations are less likely to benefit with ICI. Therefore, it has become increasingly crucial to perform molecular testing for oncogenic driver alterations in early-stage disease. While we continue our efforts to increase molecular testing rates in advanced NSCLC, efforts need to be made to promote NGS testing across all stages. Ongoing studies of neoadjuvant targeted therapies such as NeoADAURA (NCT04351555) and LCMC4 (NCT04712877) are likely to make molecular testing the standard of care in early-stage NSCLC.

*Do patients with very early-stage disease benefit from ICI?* Most perioperative ICI based trials included patients with at least stage IB (>4cm) or higher disease. These inclusion criteria were extrapolated from prior chemotherapy trials, where no benefit was seen in stage IA disease. While stage IA disease predicts better outcomes, 5-year OS is only about 60% and recurrence rates are between 20% and 40% [78]. In comparison, 5-year OS in stage I breast or colorectal cancer isover 90%[79, 80]. This presents an opportunity to test ICI therapy as a means of providing long-term tumor surveillance and improve survival in very early stages of NSCLC.

## Conclusion

We have witnessed monumental improvement in survival rates of advanced NSCLC in the past few years. Now, perioperative immunotherapy, especially neoadjuvant chemoimmunotherapy, has changed the decades long standard of care for patients with resectable NSCLC. As immunotherapy use in the curative setting soars, a challenge will be to avoid over-treatment and exposure to unnecessary toxicity. With the number of ongoing clinical trials in this space, the treatment paradigm will continue to go through a transformation in the coming years as we witnessed in advanced NSCLC. These strategies, combined with other measures such as improved screening to diagnose more cancers in the early stage, better response and residual disease detection assays, and improved surgical resectability, hold great potential to improve cure rates and overall survival in resectable NSCLC.
